# Promoter activity of ORF-less gene cassettes isolated from the oral metagenome

**DOI:** 10.1038/s41598-019-44640-2

**Published:** 2019-06-10

**Authors:** Supathep Tansirichaiya, Peter Mullany, Adam P. Roberts

**Affiliations:** 1 Department of Microbial Diseases, University College London, Eastman Dental Institute, 256 Gray’s Inn Road, London, WC1X 8LD UK; 2 Department of Tropical Disease Biology, Liverpool School of Tropical Medicine, Pembroke Place, Liverpool, L3 5QA UK; 3 Present Address: Department of Clinical Dentistry, Faculty of Health Sciences, UiT the Arctic University of Norway, Tromsø, Norway

**Keywords:** Bacterial genetics, Bacterial genetics

## Abstract

Integrons are genetic elements consisting of a functional platform for recombination and expression of gene cassettes (GCs). GCs usually carry promoter-less open reading frames (ORFs), encoding proteins with various functions including antibiotic resistance. The transcription of GCs relies mainly on a cassette promoter (P_C_), located upstream of an array of GCs. Some integron GCs, called ORF-less GCs, contain no identifiable ORF with a small number shown to be involved in antisense mRNA mediated gene regulation. In this study, the promoter activity of ORF-less GCs, previously recovered from the oral metagenome, was verified by cloning them upstream of a *gusA* reporter, proving they can function as a promoter, presumably allowing bacteria to adapt to multiple stresses within the complex physico-chemical environment of the human oral cavity. A bi-directional promoter detection system was also developed allowing direct identification of clones with promoter-containing GCs on agar plates. Novel promoter-containing GCs were identified from the human oral metagenomic DNA using this construct, called pBiDiPD. This is the first demonstration and detection of promoter activity of ORF-less GCs from *Treponema* bacteria and the development of an agar plate-based detection system will enable similar studies in other environments.

## Introduction

**Figure 1:**
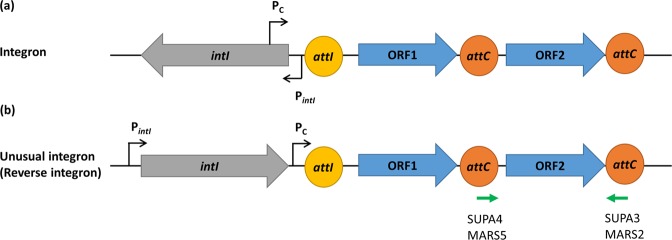
A generalised structure of (**a**) usual integrons and (**b**) unusual, or reverse integrons. The green arrows indicate the primer binding sites on the unusual integron structure of *T. denticola*. The grey and blue open arrowed boxes represent integrase gene (*intI*) and the open reading frames (ORFs), respectively, pointing in the direction of transcription. The promoters, P_*intI*_ and P_C_, were represented by black arrows. The recombination sites, *attI* and *attC*, were represented by yellow and orange circles, respectively.

Integrons are bacterial genetic elements able to integrate and express genes present on gene cassettes (GCs)^1–3^. They consist of two main components; a functional platform and a variable array of GCs. The functional platform, located on the 5′ end of an integron, consists of an integrase gene (*intI)*, and its promoter (P_*intI*_), an *attI* recombination site and a constitutive cassette promoter (P_C_) for the expression of GCs^4^. IntI is a site-specific tyrosine integrase that catalyses the insertion and excision of GCs via recombination mainly at *attI* and the *attC*, the latter located on circularised GCs. The integrase gene; *intI*, is normally transcribed in the opposite direction to GCs within an integron (Fig. 1a). However, some integrons have integrase genes transcribed in the same directions as their GCs. These are called unusual integrons or reverse integrons (Fig. 1b), and have been identified in *Treponema denticola*, *Acinetobacter baumannii, Chlorobium phaeobacteroides* and *Blastopirellula marina*^5,6^.

The second part of an integron is an array of GCs. Each usually contains a single promoterless open reading frame (ORF) and an *attC* recombination site^7^. The proteins encoded by GCs are diverse in which most GCs are either have no known homologues in the database or predicted to encode hypothetical proteins, while the remaining showed homology to proteins associated with antibiotic resistance, virulence, metabolism and etc.^2,8^. When a GC is excised from integron, it forms a non-replicative mobile genetic element, which can be a substrate for integrase mediated recombination between *attI* (on the integrons) and *attC* (on the circular GC). This directionality of recombination is favoured over *attC*:*attC* recombination, resulting in the usual insertion of a newly integrated GC immediately next to the P_C_ promoter in the first position of the GC array, ensuring maximal expression^9–11^.

The expression of integron integrases is controlled via the SOS response; there is a LexA-binding site located in the P_*intI*_^12^. In the absence of stress, the transcriptional repressor LexA binds to P_*intL*_ and prevents the transcription of *intI*. The SOS response is activated upon the accumulation of single-strand DNA (ssDNA), generated during DNA damage, DNA repair, transformation, conjugation and certain antibiotic exposure e.g. trimethoprim and fluoroquinolones^13–15^. RecA recognises ssDNA and polymerises into RecA nucleofilaments, which induce autocleavage of LexA, releasing P_*intI*_ from repression and allowing *intI* transcription^12,16^. By controlling the expression of IntI, bacteria can reshuffle their GCs at the precise moments of need (stress), generating genetic diversity and rapid adaptation to selective pressures, thereby avoiding accumulation of random recombination events that could be deleterious to the host cell^17–19^.

As most of the GCs do not contain a promoter, their expression usually relies on the P_C_ promoter. The level of expression of GCs varies depending on the distance from P_C_, as the strength of expression decreases when GCs are located further from P_C_^20^. This ensures that a recently acquired GC will be immediately expressed. There are also some GCs that contain their own promoters, ensuring constitutive expression of their genes regardless of the P_C_ promoter and their position within the integron array; examples include *cmlA1* (chloramphenicol resistance), *qnrVC1* (quinolone resistance), *ere(A)* (erythromycin resistance) and many of the GCs encoding toxin-antitoxin (TA) systems^21–24^.

Integron GCs have been identified from environments such as soils, marine sediments, seawater and more recently from human oral metagenomes^25–29^. In our previous study on the detection of integron GCs in the human oral metagenome, we found 13 ORF-less GCs out of 63 identified GCs (20%)^29^. ORF-less GCs have been shown to encode regulatory RNAs, for example the trans-acting small RNA (sRNA)-Xcc1, encoded by the ORF-less GC of a *Xanthomonas campestris* pv. *campestris* integron, which is involved in regulation of virulence^30^. Whilst promoter activity of ORF-less GCs has been discussed, this has not been experimentally demonstrated^8^.

In this study, we performed *in silico* analysis to identify promoter sequences in the GCs identified in our previous study on the oral metagenome. Promoter activity was experimentally determined by cloning the selected GCs upstream of the *gusA* reporter gene and measuring β-glucuronidase enzyme activity. Furthermore, we devised a GC-based promoter detection strategy utilising PCR and subsequent cloning between divergently orientated reporter genes. With this system, the successful cloning of amplicons from promoter-containing GCs can be visualised directly on agar plates, allowing the direct isolation of GC PCR amplicons with promoter activity from metagenomic DNA.

## Results

### Determination of promoter activity of the ORF-less GCs using the β-glucuronidase assay

Among 63 GCs previously identified from human oral metagenomic DNA, 13 were predicted to be ORF-less GCs^29^. Five GCs were chosen for experimental expression analysis. GC TMB4 (amplified with primers targeting *intI* and *attC*) was selected as it is ORF-less and located in the first position of the integron array^29^. ORF-less GCs MMU23 and MMB37 were selected as they had the highest overall score predicted by BPROM promoter prediction software (Supplementary Table S1). Finally, GCs SSU17 and MMB3 were selected as controls, to represent GCs with an ORF. In this study, we have defined the sense strand as the same strand containing the P_C_ promoter (Fig. 1).

**Figure 2:**
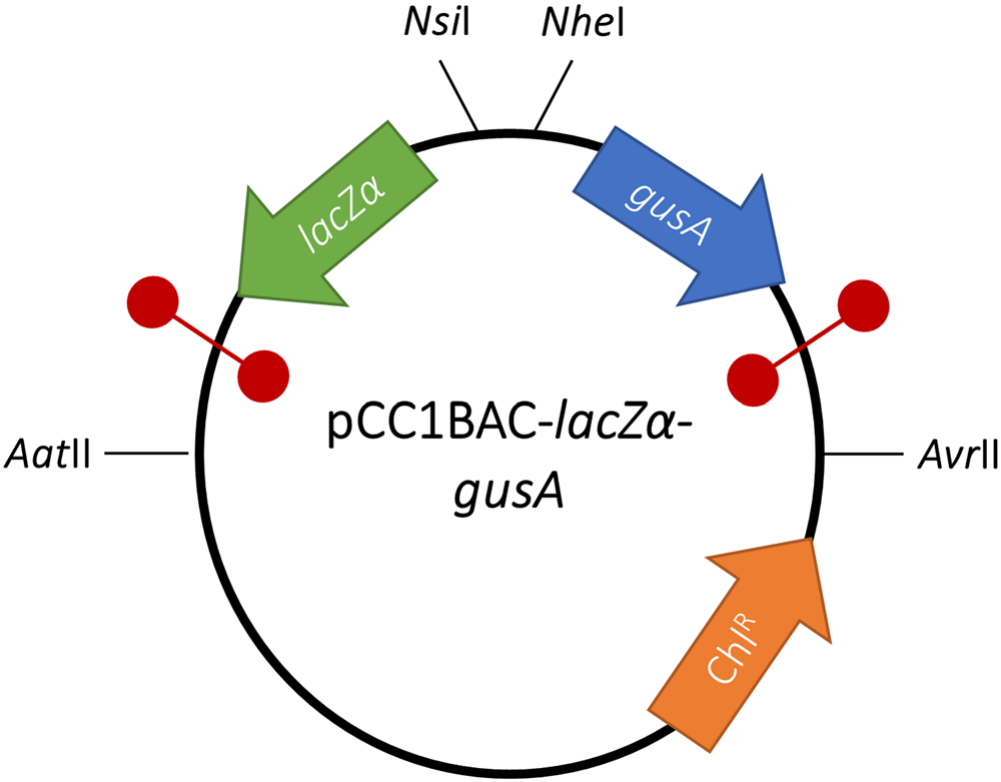
The structure of pCC1BAC-*lacZα*-*gusA* plasmid. The green, blue and orange open arrowed boxes represent *lacZα, gusA* and chloramphenicol resistance gene, respectively, pointing in the direction of transcription. The black lines indicate the position of restriction sites on the plasmid. The red circles indicate bidirectional transcriptional terminators.

As BPROM predicted putative promoter sequences on both strands, promoter activity of the selected GCs was determined by directionally cloning upstream of a promoterless β-glucuronidase (*gusA*) gene on pCC1BAC-*lacZα*-*gusA* (Fig. 2) in both directions. As the selected GCs were likely derived from *Treponema* spp., two experimentally verified *T. denticola* promoters, P_TdTro_ and P_Fla_, were also included as controls showing that *T. denticola* promoters can be recognised in our *E. coli* host^31,32^. P_Fla_ and P_Tdtro_ were selected as they rely on different sigma factors. P_Tdtro_ is recognised by sigma factor 70 (σ^70^) that is responsible for the transcription of most genes during growth in both *E. coli* and *Treponema* spp.^31,33^, while P_Fla_ is recognised by sigma factor 28 (σ^28^), involved in the expression of flagella-related genes in motile bacteria^32,34^. This will determine the limitations of our assay in recognising promoters associated with different types of sigma factors.

**Figure 3:**
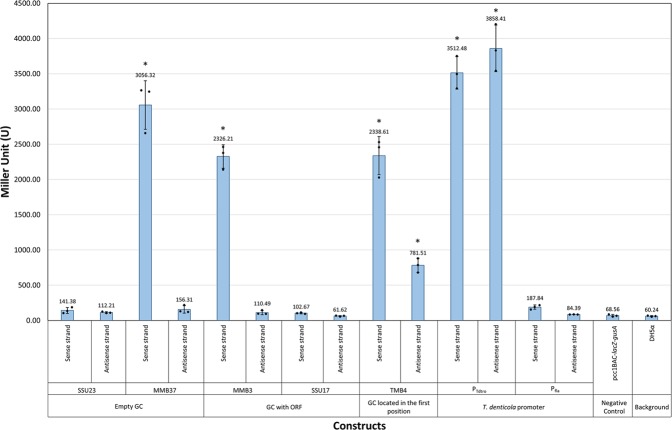
The promoter activity of ORF-less GCs estimated by β-glucuronidase enzyme assays. Error bars indicate the standard errors of the means from three replicates. The scatter plots indicate the result from each replicate and the average Miller units for each construct were shown above the bars. The asterisks (*) indicate the constructs were statistically significantly different from the negative control group (pCC1BAC-*lacZα-gusA*) with the p-value < 0.05 by using ordinary one-way ANOVA followed by Dunnett’s multiple comparison tests.

The results showed that MMB37 and MMB3 had promoter activity on one strand, while MMU23 and SSU17 had no promoter activity, compared to the negative control, while the TMB4 GC showed promoter activities on both strands (Fig. 3). The P_Tdtro_ from *T. denticola* showed strong promoter activity on both sense and antisense strands, while P_Fla_ showed no promoter activity, suggesting that σ^70^ promoters, but not σ^28^ promoters, from *T. denticola* can be recognised by *E. coli*.

### Determination of synergy between P_C_ and the ORF-less promoter GC in first position using the β-glucuronidase assay

Previously, coupling P_C_ with another promoter has been shown to significantly increase the expression of GCs, such as the presence of a second promoter (P_2_) downstreaming from P_C_ and the presence of two P_C_ in class 2 integrons (P_C2A_ and P_C2B_), could result in a significantly higher expression of GCs^35–37^. As the promoter activities of the TMB4 GC, located in the first position of GC array, were confirmed in the previous section, another two constructs (TMB4 P_C_ promoter and TMB4 P_C_-GC constructs) were therefore constructed to determine the synergy effect between P_C_ and the ORF-less promoter GC. As the TMB4 P_C_ promoter was not identical to the P_C_ of *T. denticola* integron^38^, the P_C_ of another integron GC; TMB1^29^, which was identical to it^39^, was also included.

**Figure 4:**
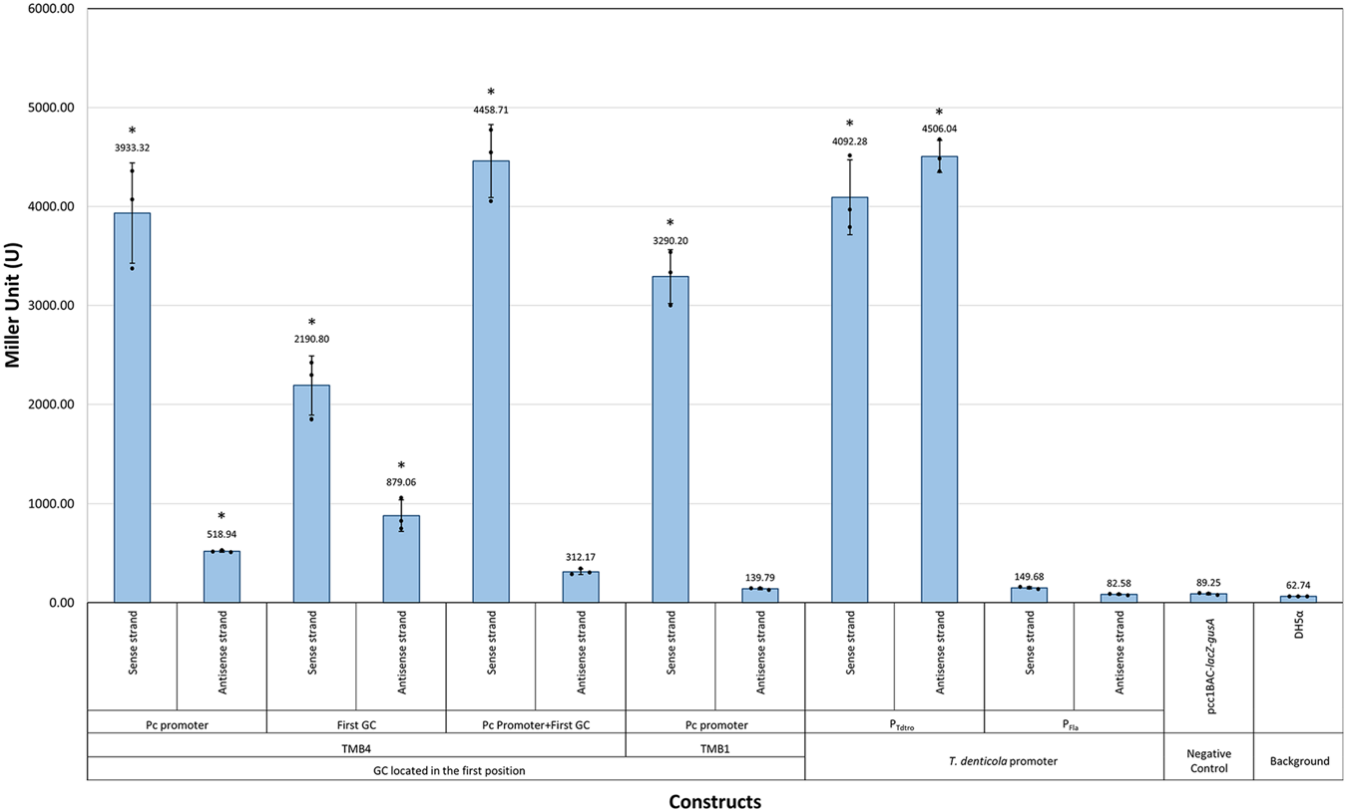
The synergy effect between P_C_ and the TMB4 ORF-less GC in the first position estimated by β-glucuronidase enzyme assays. Error bars indicate the standard errors of the means from three replicates. The scatter plots indicate the result from each replicate and the average Miller units for each construct were shown above the bars. The asterisks (*) indicate the constructs were statistically significantly different from the negative control group (pCC1BAC-*lacZα-gusA*) with the p-value < 0.05 by using ordinary one-way ANOVA followed by Dunnett’s multiple comparison tests.

The results show that the TMB4 P_C_ and TMB4 P_C_-GC showed promoter activities on both directions. However, coupling promoter GC in the first position (TMB4 P_C_-GC) did not significantly increase the expression of reporter genes, compared to the presence of only TMB4 P_C_ (*p*-value > 0.99 by using ordinary one-way ANOVA followed by Bonferroni’s post-hoc) (Fig. 4). As the P_C_ promoter sequences on TMB1 and TMB4 samples were different at several nucleotides, it was shown that TMB4-P_C_ had higher promoter activities than the TMB1-P_C_ in both directions (Fig. 4).

### Detection of promoter-containing GCs from oral metagenome

**Figure 5:**
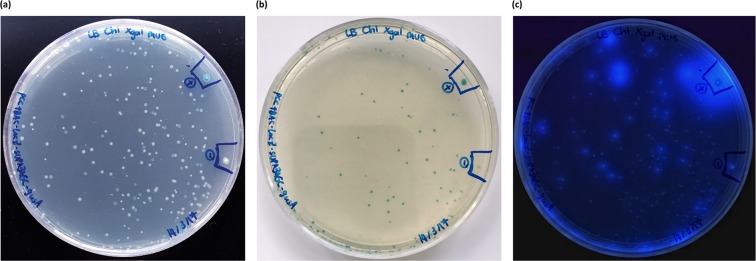
The detection of the integron GCs by using pBiDiPD. (**a**) pBiDiPD Transformants on LB agar supplemented with chloramphenicol, X-gal/IPTG and 4-methylumbelliferyl β-D-glucuronide (MUG), (**b**) Blue-white screening to detect for the clones with promoter activity on the antisense strand, (**c**) Exposing the colonies under the UV light to detect clones with promoter activity on the sense strand. The positive (+) and negative (−) colonies were the *E. coli* containing pCC1BAC-*lacZα*-TMB4-P_C_-*gusA* (with experimentally proven promoter activities on either strand of DNA and pCC1BAC-*lacZα*-*gusA* (no promoter activity), respectively.

The pCC1BAC*-lacZα-gusA* plasmid, developed for the above enzymatic assay, had the potential to be used in an agar plate-based detection strategy to detect amplified integron GCs with promoter activity on either strand of DNA. This construct is called the Bi-Directional Promoter Detection plasmid (pBiDiPD). To verify the utility of pBiDiPD, and also to detect novel GCs containing promoter sequences in the human oral metagenome, integron GCs were amplified with SUPA4-*Nsi*I/SUPA3-*Nhe*I and MARS5-*Nsi*I/MARS2-*Nhe*I primers^29^, and cloned into pBiDiPD. The clones with GCs containing a promoter on the sense strand showed blue fluorescence when visualised under UV light, reflecting the activity of β-glucuronidase enzymes catalysing MUG to yield the blue-fluorescent 4-methylumbelliferyl. Clones with promoter activity on the antisense strand resulted in blue colonies as a result of β-galactosidase enzymes catalysing X-Gal into a blue insoluble pigment 5,5′-dibromo-4,4′-dichloro-indigo (Fig. 5).

After screening clones from both amplicon libraries (amplified with SUPA3-SUPA4 and MARS2-MARS5 primers), described in materials and methods, 23 different GCs with promoter activities were identified (Table 1 and Supplementary Table S2). Fourteen of these were similar to the GCs identified in the previous study with >86% nucleotide identity^29^. Among the recovered promoter-containing GCs, 9 out of 23 were novel including sample SSU-Pro-20, SSU-Pro-27, SSU-Pro-32, SSU-Pro-46, SSU-Pro-65, MMU-Pro-5, MMU-Pro-24, and MMU-Pro-53.

**Table 1:** Summary of human oral integron GCs containing promoter sequences detected by pBiDiPD.

Characteristics			Number of unique gene cassettes		
			Primers SUPA3-SUPA4	Primers MARS5-MARS2	Total
Sequence analysis of GC amplicons	Number of screened GC amplicons		62	58	120
	Discarded amplicons	Chimeric amplicons	23	11	34
		Incompete GCs	13	2	15
	Number of GCs followed all criteria		26	45	71
**Number of unique GCs with promoter activities**			12	11	23
Homologues	GCs found in previous study^29^		7	7	14
	Novel GCs		5	4	9
Orientations and Identities of GCs	ORF-less GCs		1	6	7
	Toxin-Antitoxin GCs		10	3	13
	Others		—	3	3
Directions of promoters	Sense strand (*gusA*)		11	3	14
	Antisense strand (*lacZ*)		—	3	3
	Both directions (*gusA* and *lacZ*)		1	5	6

The GCs can be categorised into two groups, one predicted to encode toxin-antitoxin systems in 12 out of 23 GCs, including plasmid stabilization protein (toxin)-prevent-host-death protein (antitoxin), BrnT (toxin)-BrnA (antitoxin), VapC (toxin)-AbrB/MazE/SpoVT family protein (antitoxin), RelE/ParE family (toxin)-XRE transcriptional regulator (antitoxin). The second group contained ORF-less GCs, which could be found in 7 samples, all reported in the previous study, except sample MMU-Pro-53. Most of the samples (14 out of 23 GCs) showed the promoter activity only on the sense strand. Samples with promoter activity only on the antisense strand were MMU-Pro-6, MMU-Pro-63, and MMU-Pro-65, while 6 out of 23 GCs exhibited promoter activity on both strands.

## Discussion

Integrons are important disseminators of antimicrobial resistance genes in which more than 130 distinct GCs carrying antimicrobial resistance genes, covering most classes of antibiotics, have been identified^40,41^. Therefore, it is important to understand the diversity of GCs and how their expression is controlled. Even though ORF-less GCs have been found in the previous studies^25,28,29,42,43^, their functions have not been fully understood. Our findings here confirmed the promoter activities of ORF-less GCs from *Treponema* spp., which could be important for the expression of other GCs in integrons.

In this study, we determined promoter activity from GCs isolated by PCR from metagenomic DNA by measuring promoter activity from multiple GC containing constructs. As the ORF-less GCs were recovered from the oral metagenome, there is little information regarding the original host. Therefore, we chose to test the promoter activities by using an *E. coli* surrogate. Nucleotide sequence analysis suggested that these GCs were likely to be derived from *Treponema* spp., therefore, the ability of *E. coli* to utilise *T. denticola* promoter sequences was determined by including the experimentally verified *T. denticola* promoter, P_TdTro_^31^ which showed high activity on both sense and antisense strands, providing confidence that *E. coli* could be used. However, as no promoter activity was detected from P_Fla_, it suggested that our enzymatic assay cannot detect promoters associated with σ^28^ from *Treponema* spp., which could be due to an inability for the *E. coli* host to recognise the *Treponema* σ^28^ promoter sequence. Therefore, constructs with no promoter activity in our enzymatic assay could also carry *Treponema* promoters associated with other sigma factors that cannot be recognised by the *E. coli* host like the σ^28^ P_Fla_ promoter.

Promoter activities of the ORF-less GCs were confirmed and quantified by using a β-glucuronidase enzymatic assay. This is the first time that the promoter activity of ORF-less GCs has been demonstrated *in vitro*, as shown by the activity on the sense strand of the MMB37 and both strands of the TMB4. A study on the *Vibrio* integron, containing a 116-cassette array, showed that most of the GCs are transcribed^44^. Therefore, ORF-less GCs could be responsible for transcription of the other GCs not transcribed by P_C_.

For the TMB4 GC (ORF-less GC in the first position), it was initially hypothesised that the promoter could help increase the expression of the downstream GCs. The constructs of TMB4 P_C_ and TMB4 P_C_ + GC were therefore included in the assay to determine whether having a promoter GC at the first position could help promote the expression of downstream GCs. However, the presence of promoter sequences in TMB4 GC did not significantly increase the expression of *gusA*. The lack of additive promoter activity can be explained by more competition for enzymes involved in transcription such as RNA polymerases (RNAP) or sigma factors to be available for transcription from each promoter, resulting in a lower transcriptional level^45^. Another, not mutually exclusive possibility is transcriptional interference (TI) between the four promoters on the TMB4 Pc + GC construct. We have experimentally shown promoter activity of TMB4 P_C_ and TMB4 GC constructs on both strands, indicating convergent TI is a possibility.

In integrons, P_C_ is in *intI*, which is convergent to the integron integrase promoter P_*IntI*_ downstream (Fig. 1), resulting in TI. The TI between P_C_ and P_*IntI*_ has been shown to control the expression of integrase and the subsequent recombination of GCs. The weaker strength of P_C_ could result in higher expression of integrase, which increases recombination of GCs^46,47^.

Promoter activity from the antisense strand in the first position of an integron, could potentially increase the expression of *intI* in integrons depending on the patterns of TI. Convergent expression between P_C_ and a reverse GC promoter in an integron could relieve the repression of the P_*intI*_ due to TI between P_*intI*_ and P_C_. This could increase recombination at *attI* due to more integrase being produced catalysing the recruitment of a new GC at the first position (Supplementary Fig. 1). With reverse integrons, a relationship with increased expression of the integrase with a promoter cassette in the first position is more difficult to envisage. However, based on our enzymatic assay, it was shown that, in the presence of TMB4 GC, there was a decrease in antisense promoter activity in TMB4 (compared to TMB4 P_C_), therefore, it could result in lower TI with P_*intI*_, resulting in a higher expression of *intI* and subsequent catalysis of recruitment of a new GC to the first position (Supplementary Fig. 2).

Previously, only a few of ORF-less GCs have been reported in the first position of integrons. For example, only 1 out of 42 GCs and 1 out of 5 GCs in the first GC position were identified as ORF-less GCs in marine^28^ and oral metagenomes^29^, respectively. This could be because when ORF-less GCs with antisense promoters are located in the first position, integrase expression and the recombination events are increased, leading to an insertion of new GCs into the first position. When the new GC is inserted in the first position, it will push the promoter GC further down the array, which will lead to previous, lower levels of integrase expression, preserving the new GC in the first position.

As integrons contain highly diverse GCs that can be shuffled to different position within their GC arrays, ORF-less GCs, other than TMB4 GC, could be shuffled to the first position next to P_C_. Therefore, there is a possibility for some ORF-less GCs act in synergy with their cognate P_C_ when they are shuffled into the first position and some, such as the one we have tested in TMB4 do not.

**Figure 6:**
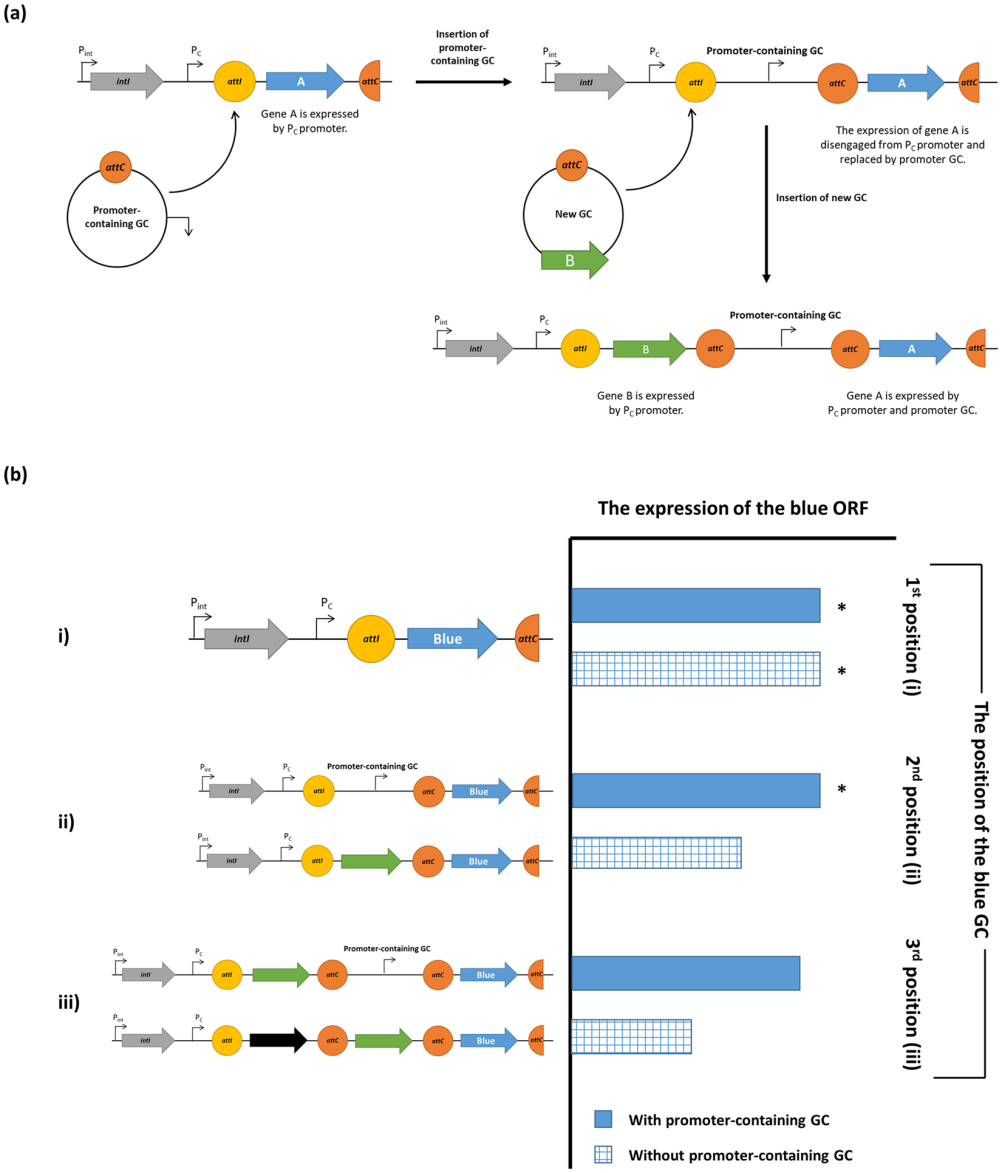
The proposed genetic clutch. (**a**) When a promoter-containing GC inserts into the first position, it can act as a genetic clutch by disengaging the original first GC (blue arrow) from P_C_ promoter and replaced with the one on promoter GC. When a new GC (green arrow) inserts, it can be expressed by P_C_ promoter, while the blue GC is expressed by promoter-containing GC and P_C_ promoter. (**b**) The expression level of gene cassettes with and without a genetic clutch. The estimated levels of expression of the blue ORF in (i) the first, (ii) the second and (iii) the third position were shown in the bar chart. The solid bars represent the situation when promoter-containing GC was inserted upstream of the blue GC, while the gridded bars represent the situation when no promoter-containing GC was inserted. The asterisks indicate the experimentally verified expression level, suggested by the results in Fig. 4 (TMB4 P_C_ and TMB4 P_C_ + GC). The expression of the blue ORF was hypothesised to be decreased when more GCs are inserted without the presence of a promoter-containing GC as a genetic clutch (gridded bars), based on the data from previous study^49^.

The expression level of cassette genes located further down in the array normally decreases due to the formation of a stem-loop structure on mRNA at *attC* sites, which impede the progression of the ribosome^48^. It was previously shown that the level of streptomycin resistance was reduced four-fold, when the *aadA2*-containing GC was located in the second position^49^. However, our data shows that the insertion of an ORF-less, promoter-containing GC in the first position did not decrease the *gusA* expression significantly (considered as a proxy for the expression of gene(s) in the second GC), i.e. comparing the data for TMB4 P_C_ and TMB4 P_C_ + GC. Therefore, we hypothesised that promoter-containing GCs could act as a genetic clutch, where the expression of the original first GC is partially disengaged from the P_C_ promoter and replaced by the one on the ORF-less promoter containing GC (Fig. 6a). This can prevent a significant change in expression of the first GC while a new, first GC is sampled from the pool of GCs in order to adapt to an additional stress concurrent with the selective pressure requiring expression of the first GC. This system would work as a genetic clutch with the insertion of any GC containing a promoter in the same direction as P_C_, so it could be the insertion of either ORF-less GCs such as TMB4 GC, or other promoter-containing GCs such as the multiple TA-containing GCs we have identified; providing another selective advantage to retaining them and explaining their varied position within the GC array.

A genetic clutch within an integron can be of benefit to bacteria when they are exposed to multiple environmental stresses such as two different antibiotics simultaneously. The first resistance gene (green ORF in Fig. 6biii) can be expressed by the P_C_ promoter, while the second resistance gene (blue GC), located in the third position, is expressed by P_C_ and the promoter GC. Therefore, allowing bacteria to survive in the presence of both drugs.

As the other ORF-less GC MMU23 showed no promoter activity it may have other functions or carry a promoter that can be recognised in its native host but not in *E. coli*, or require other sigma factors. For the ORF-containing GC MMB3 sample, the promoter activity was found on the sense strand. This GC was predicted to carry toxin-antitoxin (TA) ORFs, including the PIN toxin and ribbon-helix-helix antitoxin domains, which were shown to contain their own promoter. Sample SSU17 and MMU23, which showed no promoter activity, can be considered as a control; illustrating that not all of GCs amplified from the oral metagenome exhibited promoter activity within our assay.

The pCC1BAC-*lacZα*-*gusA* plasmid, developed for the enzymatic assay, also had potential to be used for the detection of promoter activity in either direction from GCs. To verify the application of pCC1BAC-*lacZα*-*gusA* plasmids as promoter detection system, integron GCs were amplified from the human saliva metagenome by using SUPA3-SUPA4 and MARS2-MARS5 primers, which were the only two primer pairs that were verified for the amplification of integron GCs from the oral metagenome^29^. After cloning the amplified GCs between both reporter genes, two groups of GCs were identified with promoter activities: ORF-less GCs and TA-containing GCs. By detecting 7 clones containing ORF-less GCs with promoter activity it further supported that one of the functions of ORF-less GCs in integrons is to provide promoter activities.

TA-containing GCs are abundant in sedentary chromosomal integrons (SCIs), which were suggested to have a role in preventing random deletion of GCs and stabilising the large arrays SCIs^23,50–52^. TA systems normally encode a stable toxin and a labile antitoxin^53^, therefore TA cassettes have to carry their own promoters to ensure their expression. These were found in SCIs of *Treponema* spp., such as the HicA-HicB TA-containing GC in the fourth position within the GC array (Accession number NC_002967) in the SCI from *T. denticola*^38^. As most of the GCs amplified with our primers were homologous with *Treponema* spp., these TA-containing GCs should be present in our oral metagenome and were detected by our pBiDiPD based on their promoter activities.

Two of the GCs, SSU-Pro-9 and MMU-Pro-18, were similar to the MMB3 and MMB37 GCs, respectively, which were shown by the β-glucuronidase enzyme assay to have promoter activity on the sense strand. The phenotypes of SSU-Pro-9 and MMU-Pro-18 colonies also showed only a blue fluorescence phenotype, reflecting the promoter activity on the sense strand, which corresponded with the enzymatic assay results of MMB3 and MMB37.

To summarise, the promoter activities of the *Treponema* ORF-less integron GCs were experimentally demonstrated by using a robust β-glucuronidase enzyme assay, confirming that one of the functions of ORF-less GCs is to provide promoters for the expression of ORF containing GCs, in addition to expression from P_C_. This could be extended to ORF-less GCs from other bacteria, which should be determined further. The dual reporter plasmid; pBiDiPD, was developed for the direct visualisation of clones containing gene cassettes with promoter activity on agar plates. This can be applied as a detection system for promoter activity for any other DNA fragments.

## Materials and Methods

### *In silico* analysis of the human oral cavity gene cassettes and the construction of pCC1BAC-*lacZα-*GC-*gusA* constructs

All of the ORF-less GCs and some of the GCs containing ORFs, identified in the previous study^29^, were predicted for putative promoter sequences by using the web-based software BPROM in the Softberry package^39^.

### Construction of pUC19-GC-*gusA* and pCC1BAC-*lacZα-*GC-*gusA* constructs

To determine the promoter activity of the selected GCs, the constructs were initially cloned into the EcoRI and KpnI restriction sites on pUC19-P*tet*(M)-*gusA* plasmid^54^. The selected GCs were amplified from the pGEM-T easy vector containing the GC amplicon from a previous study^29^, as shown in Supplementary Fig. 3, by using primer listed in Supplementary Table S3.

Due to a significant difference in the plasmid copy number in some constructs of the pUC19-GC-*gusA*, new constructs were prepared based on a low copy number CopyControl™ pCC1BAC™ vector (Epicenter, UK) as it will be maintained in *E. coli* cell as one plasmid per cell and enable us to control the plasmid copy number to be similar between each construct. The construct was designed to contain two reporter genes, β-galactosidase *lacZα* and β-glucuronidase *gusA* genes (Fig. 2 and Supplementary Fig. 4). As *lacZα* on pCC1BAC contained T7 promoter sequence, it was first deleted by using Q5® Site-Directed Mutagenesis Kit (New England Biolabs, UK). The backbone of pCC1BAC was amplified with pCC1BAC-del*LacZ-*F1 and pCC1BAC-del*LacZ-*R1, and the amplified products were treated with a Kinase-Ligase-DpnI (KLD) enzyme mix, following the instructions from the manufacturer. The KLD-treated product was then transformed into *E. coli* α-Select Silver Efficiency competent cells (Bioline, UK) following the instructions from the manufacturer. The pCC1BAC-del*LacZ* plasmid was then extracted from *E. coli* by using QIAprep Spin Miniprep Kit (Qiagen, UK), following the manufacturer’s instructions.

The *lacZα* reporter gene was amplified from the pUC19 vector (New England Biolabs, UK) with *LacZ-*F1 and *LacZ-*R1 primers. For *gusA* reporter gene, it was amplified from pUC19-P*tet*(M)-*gusA* with *gusA-F1* and *gusA-R1* primers. A bidirectional terminator, modified from *lux* operon, was added to *LacZ-*F1 and *gusA*-R1 primers, resulting in two bi-directional terminators flanking the *lacZα-gusA* reporter genes^55^. This was done to prevent transcriptional read-through from the promoter in the plasmid backbone and to also prevent promoters from the inserts interfering with the expression of genes on the plasmid backbone. The *lacZα* and *gusA* amplicons were digested with NsiI restriction enzymes (New England Biolabs, UK) and ligated together by using T4 DNA ligase (New England Biolabs, UK). The *lacZα-gusA* ligated product was directionally cloned into the pCC1BAC-del*LacZ* plasmid by digesting them with AatII and AvrII restriction enzymes and ligated together, resulting in pCC1BAC-*lacZα-gusA* plasmid.

The selected GCs were amplified from each pUC19-GC-*gusA* constructs by using primer listed in Supplementary Table S3. The amplicons were double digested with *Nsi*I and *Nhe*I and directionally cloned into a pre-digested pCC1BAC-*lacZα-gusA* plasmid, then transformed into *E. coli* α-Select Silver Efficiency competent cells.

### Determination of β-glucuronidase enzymatic activity

The β-glucuronidase enzymatic assay was performed to measure the promoter activity based on the expression of *gusA*, following the protocol described previously with some modifications^56^. The overnight cultures of *E. coli* containing the reporter constructs were prepared in LB broth supplemented with 12.5 µg/mL chloramphenicol. The OD_600_ of each overnight culture was measured. An aliquot of 1 mL of the overnight culture was centrifuged at 3000 × *g* for 10 min and discarded the supernatant. The cell pellets were incubated at −70 °C for 1 hr and resuspended in 800 µl of pH 7 Z buffer (50 mM 2-mercaptoethanol, 40 mM NaH_2_PO_4_·H_2_O, 60 mM Na_2_HPO_4_·7H_2_O, 10 mM KCl, and 1 mM MgSO_4_·7H_2_O) and 8 µl of toluene. The mixture was transferred to a 2 ml cryotube containing glass beads (150–212 μm in diameter) (Sigma, UK) and vortexed twice for 5 min each with an incubation on ice for 1 min in between. The glass beads were then removed by centrifugation at 3000 × *g* for 3 min. One-hundred microliters of cell lysate were mixed with 700 µl of Z-buffer, then incubated at 37 °C for 5 min. One-hundred sixty microliters of 6 mM ρ-nitrophenyl-β-D-glucuronide (PNPG) was then added to the reaction and incubated at 37 °C for 5 min. The reactions were stopped by adding 400 µl of 1 M Na_2_CO_3_ and centrifuged at 3000 × *g* for 10 min to remove cell debris and glass beads. The absorbance of the supernatant was measured with a spectrophotometer at the wavelength of 405 nm. Three biological replicates of the β-glucuronidase enzymatic assay were performed. The β-glucuronidase Miller units were calculated from^57^

$$\frac{{A}_{405}\times 1000}{O{D}_{600}\times {time}\,({\rm{\min }})\times 1.25\times {\rm{volume}}({\rm{mL}})}\,.$$

### Statistical analysis

The average and standard deviation of β-glucuronidase concentration were calculated from three biological replicates, which were used for the columns and error bars in Fig. 3, respectively. The statistical comparisons between the negative control (pCC1BAC-*lacZ-gusA*) to the other constructs were performed by using ordinary one-way ANOVA with either Dunnett’s post-hoc test (to compare each construct with a negative control) or Bonferroni’s post-hoc test (to compare constructs between themselves). The groups with statistically significantly difference from the control had the *p-*value of less than 0.05.

### Recovery of promoter-containing GCs from the human oral metagenome

The integron GCs were amplified from the human oral metagenome by using as described previously with SUPA4-NsiI-SUPA4-NheI and MARS5-NsiI-MARS2-NheI primers^29^. The human oral metagenomic DNA was previously extracted from the saliva samples collected from 11 volunteers in the Department of Microbial Diseases, UCL Eastman Dental Institute^29^. The ethical approval for the collection and uses of saliva samples was obtained from University College London (UCL) Ethics Committee (Project number 5017/001). The written informed consents were obtained from all volunteers prior to the collection of saliva samples. All procedures performed in this study were in accordance with the ethical guidelines and regulations from the UCL Ethics Committee.

The amplified products were purified and digested with NsiI and NheI and ligated with the pre-digested pCC1BAC-*lacZα-gusA* plasmid. The ligated products were transformed into *E. coli* α-Select Silver Efficiency competent cells by heat shock. Cells were spread on LB agar supplemented with 12.5 µg/mL chloramphenicol, 80 µg/mL X-gal, 50 µM IPTG, and 70 µg/mL 4-methylumbelliferyl-β-D-glucuronide (MUG). After incubation at 37 °C for 18 hr, the colonies with β-galactosidase activity from *lacZ* was detected by blue-white screening on the agar plate, and the β-glucuronidase activity from *gusA* was visualisation under UV light. Colonies exhibiting either activity were selected and subcultured on fresh agar plates. The inserts were amplified by colony PCR using *lacZ-*F2 and *gusA*-*F2* primers and sequenced by sequencing service from Genewiz (Genewiz, UK).

### Sequence analysis and nomenclature of promoter-containing GC amplicons

DNA sequences were visualised and analysed by using BioEdit version 7.2.0 (http://www.mbio.ncsu.edu/bioedit/bioedit.html). The contigs from sequencing reactions were combined by using CAP contig function in the software^58^. The sequences were then matched to the nucleotide and protein database by using BlastN and BlastX (version 2.8.0) from the National Centre for Biotechnology Information (NCBI), respectively^59^. ORF finder (https://www.ncbi.nlm.nih.gov/orffinder) and BlastP (version 2.8.0) were used for the identification of ORFs in each GC^60^. The criteria for the sequence analysis of integron GC were the same as described in the previous study^29^. Artefactual PCRs were discounted by detecting the consensus R′ (1 R) core sites [GTTN_1_N_2_N_3_N_4_] and the complementary R″ (1 L) core sites [N′_4_N′_3_N′_2_N′_1_AAC] of *attC* located downstream from the *attC* forward primers and upstream from the *attC* reverse primers, respectively, where N and N′ are complmentary nucleotides and the number indicated their positions (Supplementary Table S4)^29,61,62^. While the conserved complimentary GTT/AAC triplet in each core site was essential, we accepted 50% non-complementarity within N_1_–N_4_. Two additional criteria for the verification of GCs detected with pCC1BAC*-lacZα-gusA* were included. Any clones containing incomplete GCs, caused by digestion at internal NsiI and NheI restriction sites on the GCs, were excluded from the dataset. Also chimeric inserts, which were the ligation products between digested amplicons, were also excluded.

The promoter-containing GCs were named as described in the previous study^29^. The first and second letters represented the forward primer and reverse primer used in the amplification. The third letter represents the source of the human oral metagenomic DNA which is U for the United Kingdom. This was followed by term “Pro”, indicating the presence of promoter activity, and the number of the clone. For instance, SSU-Pro-1 stands for the first clone amplified from the UK oral metagenome by using SUPA3 and SUPA4 primers. The sequences of these GCs were deposited in the DNA database with the accession number from MH536747 to MH536769.

## Supplementary information

Supplementary information
