# Supplementary material for: Promoter activity of ORF-less gene cassettes isolated from the oral metagenome
Source: Sci Rep. 2019 Jun 10;9:8388. doi: 10.1038/s41598-019-44640-2 (PMC6557892; doi:10.1038/s41598-019-44640-2)
Supplement: Supplementary file 1 — Supplementary information [file 41598_2019_44640_MOESM1_ESM.pdf]

Supporting Information

Promoter activity of ORF-less gene cassettes isolated from the oral metagenome

Supathep Tansirichaiya[1,#a], Peter Mullany[1], Adam P. Roberts[1,2]*

[1]Department of Microbial Diseases, University College London, Eastman Dental Institute, 256 Gray's Inn Road, London, WC1X 8LD, UK.

[2]Liverpool School of Tropical Medicine, Pembroke Place, Liverpool, L3 5QA, UK

*Corresponding author; Liverpool School of Tropical Medicine, Pembroke Place, Liverpool, L3 5QA, UK

T: +44(0)151 705 3247. E: Adam.Roberts@lstmed.ac.uk

[#a]Current Address: Department of Clinical Dentistry, Faculty of Health Sciences, UiT the Arctic University of Norway, Tromsø, Norway

Supplementary Table S1. The putative promoters for ORF-less GCs and GCs with an ORF (SSU17 and MMB3) predicted using BPROM.

Clones	Strand	-10 box	-35 box	Score		
				-10 box	-35 box	Linear discriminant function (LDF)* (Overall score)
TMB4	+	AGGTATAAT	ATAAGA	89	-10	9.78
	-	CATTATTTT	TTGACA	41	66	7.60
SSU9	+	AATTATAAT	TAAAAA	74	0	7.04
	-	TAGTATAAT	TTTATT	80	34	7.11
MMU2	+	AATTATAAT	TTAAAA	74	37	8.36
	-	TAGTATAAT	TTTATT	80	34	8.90
MMU11	+	ATGTAAAAT	TTGCTG	75	47	11.34
	+	AACTATACT	AGGAAA	59	-7	5.99
	-	AAATAAAAT	TTTTCA	56	34	6.96
	-	CTATAAATT	TTTCAA	44	36	3.24
MMU19	+	AGGTATAAT	TAGAAA	89	23	9.07
	+	TTGAAAAAT	TTGCGG	44	32	3.43
	-	TATTATAAT	TTTCCT	79	37	9.10
MMU23	+	AATTATAAT	TAAAAG	74	-6	9.84
	+	TTTTATTAT	TTGATG	72	52	6.05
	-	TATTATAAT	TTTCCT	79	37	8.66
	-	TAGTATAAT	TTTATT	80	34	8.05
MMB2	+	AATTATAAT	TATAAG	74	-2	8.71
	+	TATTATAAT	TTGATG	79	52	7.88
	-	TATTATAAT	TTTCCT	79	37	9.10
	-	TATTATAAT	TTTATT	79	34	8.84
MMB5	+	AATTATAAT	TTAAAA	74	37	8.36
	-	TAGTATAAT	TTTATT	80	34	7.95

Clones	Strand	-10 box	-35 box	Score		
				-10 box	-35 box	Linear discriminant function (LDF)* (Overall score)
MMB20	+	AATTATAAT	TAAAAG	74	-6	9.09
	-	TATTATAAT	TTTCCT	79	37	9.10
MMB32	+	TATTATAAT	TTGATG	79	52	6.28
	+	AGATATAAA	GTGTAA	39	14	4.84
	-	TATTATAAT	TTGATT	79	53	6.61
	-	TTTTATTTT	TTAAAA	52	37	5.11
MMB36	+	AATTATAAT	TTAAAA	74	37	6.94
	+	TATTATAAT	TTGATG	79	52	6.45
	-	TATTATAAT	TTTATT	79	34	7.44
	-	TTTTAAAAT	TTGACT	79	61	6.13
MMB37	+	AATTATAAT	TAAAAG	74	-6	9.11
	+	TTATATAAT	TTGATG	75	52	8.55
	-	TAGTATTAT	TTTATT	66	34	10.48
	-	TATTATAAT	TTTCCT	79	37	9.10
SSU17	+	CTTTATAAT	ATGAAT	82	25	7.80
	+	TGATAAAAT	GTGAAA	75	27	4.62
	-	TGATATAAT	TTTATT	82	34	9.34
	-	TGATTAGAT	TTTATG	21	33	5.10
MMB3	+	CTGTATATT	TTGATA	63	58	6.74
	+	ATTTATGAT	ATGAAA	65	30	5.18
	-	ATGTATTGT	TTGATG	44	52	6.64
	-	GCATATAAT	TTCTCT	65	28	4.75

* The LDF takes into account motifs found in promoters: -10 and -35 boxes, a distance between -10 and -35 boxes, and frequencies of certain nucleotides represented in transcription start sites. It can be approximated as log(<likelihood of a site being promoter>/<likelihood of a site not being promoter>)[1]

** The selected samples for the enzymatic assay are highlighted in yellow.

Supplementary Table S2. Characterisation of the human oral integron GCs containing promoter sequences detected by pBiDiPD.

Gene cassettes	Primer pair	Cassette Size (bp)	Orientation*	BlastN		BlastX				Promoter activity		Accesion number
				Closest homologue	Percentage identity (%)/ Coverage (%)	Closest homologue	ORF size (bp)	Percentage identity (%)/Coverage (%)	Accession number of the homologous proteins (BlastX)	Sense Strand (*gusA*)	Antisense strand (*lacZ*)	
SSU-Pro-7	SUPA3-SUPA4	1001	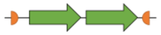	SSU22	98/95	Prevent-host-death protein (Phd_YefM antitoxin superfamily) [*Treponema vincentii*]	264	97/100	WP_006188308.1	Y	N	MH536747
						XRE family transcriptional regulator [*Treponema vincentii*]	441	98/100	WP_006188306.1			
SSU-Pro-9	SUPA3-SUPA4	834	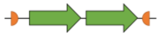	MMB3	98/99	Hypothetical protein (antitoxin, ribbon-helix-helix domain protein) [*Treponema putidum*]	246	67/100	WP_044978234.1	Y	N	MH536748
						Twitching motility protein PilT (PIN toxin domain) [*Treponema putidum*]	414	71/100	AIN93467.1			
SSU-Pro-13	SUPA3-SUPA4	855	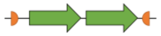	MMB39	98/99	Toxin RelE [*Treponema medium*]	357	95/100	WP_016522532.1	Y	N	MH536749
						Transcriptional regulator (Antitoxin, XRE family) [*Treponema medium*]	330	95/100	WP_016522533.1			
SSU-Pro-16	SUPA3-SUPA4	925	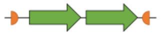	SSU28	98/100	AbrB/MazE/SpoVT family DNA-binding domain-containing protein (Antitoxin) [*Treponema putidum*]	231	96/100	WP_044979179.1	Y	N	MH536750

Gene cassettes	Primer pair	Cassette Size (bp)	Orientation*	BlastN		BlastX				Promoter activity		Accesion number
				Closest homologue	Percentage identity (%)/ Coverage (%)	Closest homologue	ORF size (bp)	Percentage identity (%)/Coverage (%)	Accession number of the homologous proteins (BlastX)	Sense Strand (*gusA*)	Antisense strand (*lacZ*)	
						Endoribonuclease MazF (Toxin) [*Treponema denticola*]	336	99/100	WP_010694033.1			
SSU-Pro-20	SUPA3-SUPA4	1263	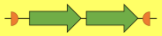	MMU28	77/42	Prevent-host-death protein (Phd_YefM antitoxin superfamily) [*Treponema sp.* JC4]	249	75/88.3	WP_009103386.1	Y	N	MH536751
						Plasmid stabilization protein (ParE toxin superfamily) [*Treponema sp. JC4]*	147	57/43.8	WP_009104800.1			
SSU-Pro-24	SUPA3-SUPA4	425	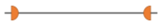	SSU9	99/100	-	-	-	-	Y	Y	MH536752
SSU-Pro-27	SUPA3-SUPA4	753	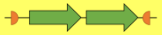	*Treponema putidum* strain OMZ 758	93/100	BrnT family toxin [*Treponema sp.*]	273	99/100	WP_002666393.1	Y	N	MH536753
						CopG family transcriptional regulator (BrnA antitoxin) [*Treponema denticola*]	288	99/100	WP_044909778.1			
SSU-Pro-32	SUPA3-SUPA4	972	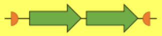	No significant similarity found.	-	RelE/ParE family toxin [*Treponema denticola*]	354	98/100	WP_002683264.1	Y	N	MH536754
						XRE family transcriptional regulator [*Treponema denticola*]	273	100/100	WP_002683262.1			

Gene cassettes	Primer pair	Cassette Size (bp)	Orientation*	BlastN		BlastX				Promoter activity		Accesion number
				Closest homologue	Percentage identity (%)/ Coverage (%)	Closest homologue	ORF size (bp)	Percentage identity (%)/Coverage (%)	Accession number of the homologous proteins (BlastX)	Sense Strand (*gusA*)	Antisense strand (*lacZ*)	
SSU-Pro-34	SUPA3-SUPA4	832	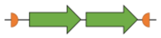	SSU5	99/100	Hypothetical protein (antitoxin, ribbon-helix-helix domain protein) [*Treponema putidum*]	246	67/100	WP_044978234.1	Y	N	MH536755
						PIN domain-containing protein [*Treponema putidum*]	414	71/100	WP_044978236.1			
SSU-Pro-39	SUPA3-SUPA4	1137	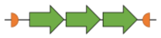	MMU25	99/99	Hypothetical protein [uncultured bacterium]	462	99/100	ANC55535.1	Y	N	MH536756
						Hypothetical protein [*Treponema maltophilum*]	213	88/100	WP_016526060.1			
						PemK/MazF family toxin [*Fibrobacter sp. UWCM*]	342	80/100	WP_022932935.1			
SSU-Pro-46	SUPA3-SUPA4	971	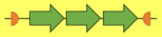	No significant similarity found	-	Hypothetical protein [*Treponema socranskii*]	267	80/100	WP_021329686.1	Y	N	MH536757
						Hypothetical protein [*Treponema socranskii*]	228	84/100	WP_021329641.1			
						DUF4160 domain-containing protein [*Treponema sp.* C6A8]	276	67/100	WP_027729334.1			
SSU-Pro-65	SUPA3-SUPA4	811	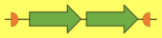	*Treponema sp.* OMZ 838	91/21	AbrB/MazE/SpoVT family DNA-binding domain-containing protein (Antitoxin) [*Treponema denticola*]	228	93/100	WP_010693782.1	Y	N	MH536758

Gene cassettes	Primer pair	Cassette Size (bp)	Orientation*	BlastN		BlastX				Promoter activity		Accesion number
				Closest homologue	Percentage identity (%)/ Coverage (%)	Closest homologue	ORF size (bp)	Percentage identity (%)/Coverage (%)	Accession number of the homologous proteins (BlastX)	Sense Strand (*gusA*)	Antisense strand (*lacZ*)	
						VapC family toxin [*Treponema denticola*]	402	93/100	WP_010693784.1			
MMU-Pro-4	MARS5-MARS2	520	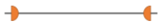	MMU2	99/100	-	-	-	-	Y	Y	MH536759
MMU-Pro-5	MARS5-MARS2	983	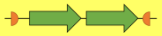	*Treponema putidum* strain OMZ 758	94/78	Prevent-host-death protein (Phd_YefM antitoxin superfamily) [*Treponema denticola*]	240	98/98.8	WP_002669519.1	Y	Y	MH536760
						RelE/StbE family addiction module toxin [*Treponema denticola*]	318	94/100	WP_002688980.1			
MMU-Pro-6	MARS5-MARS2	737	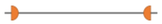	MMB36	86/100	-	-	-	-	N	Y	MH536761
MMU-Pro-18	MARS5-MARS2	634	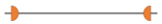	MMB37	95/100	-	-	-	-	Y	N	MH536762
MMU-Pro-22	MARS5-MARS2	431	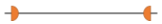	MMU19	91/100	-	-	-	-	Y	Y	MH536763
MMU-Pro-24	MARS5-MARS2	904	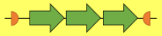	No significant similarity found	-	Universal stress protein [*Marinobacter sp.*]	348	30/54.1	WP_008177208.1	Y	Y	MH536764
						Hypothetical protein [*Methylobacter tundripaludum*]	213	79/100	WP_031438379.1			

Gene cassettes	Primer pair	Cassette Size (bp)	Orientation*	BlastN		BlastX				Promoter activity		Accesion number
				Closest homologue	Percentage identity (%)/ Coverage (%)	Closest homologue	ORF size (bp)	Percentage identity (%)/Coverage (%)	Accession number of the homologous proteins (BlastX)	Sense Strand (*gusA*)	Antisense strand (*lacZ*)	
						Prevent-host-death protein [*Treponema pedis*]	84	76/27.8	WP_024469914.1			
MMU-Pro-31	MARS5-MARS2	574	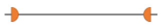	MMB5	88/70	-	-	-	-	Y	N	MH536765
MMU-Pro-48	MARS5-MARS2	817	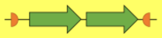	*Treponema sp.* OMZ 838	91/25	AbrB/MazE/SpoVT family DNA-binding domain-containing protein [*Treponema denticola*]	228	93/100	WP_010693782.1	Y	N	MH536766
						VapC family toxin [*Treponema denticola*]	402	93/100	WP_010693784.1			
MMU-Pro-53	MARS5-MARS2	430	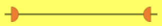	No significant similarity found	-	-	-	-	-	Y	Y	MH536767
MMU-Pro-63	MARS5-MARS2	927	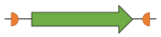	SSU8	99/99	Hypothetical protein [*Treponema denticola*]	531	98/93.7	WP_002692239.1	N	Y	MH536768
MMU-Pro-65	MARS5-MARS2	896	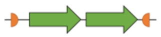	MMU27	99/100	Hypothetical protein [uncultured bacterium]	399	99/84.2	ANC55539.1	N	Y	MH536769
						Hypothetical protein [uncultured bacterium]	357	99/100	ANC55540.1			

* The orange half circles and green arrow boxes are representing *attC* sites and ORFs, respectively.

** The GCs found in this study are highlighted in yellow. Those not highlighted were also detected in Tansirichaiya et al. (2016)[2].

Supplementary Table S3; Primers used in this study

Primer name	Sequence (5'-3')[a]	Gene target	Reference
**Primers for the cloning of GCs into pUC19-*gusA* constructs**			
**SUPA4-KpnI**	CGCGCG**GGTACC**CCGCAAATGCAGGTTAAGCG	*attC* site (forward primer)	This study
**SUPA3-EcoRI**	CGCGCG**GAATTC**CAGGTTGAAGCGGGTGTTAG	*attC* site (reverse primer)	This study
**SUPA4-EcoRI**	GCGGCC**GAATTC**CCGCAAATGCAGGTTAAGCG	*attC* site (forward primer)	This study
**SUPA3-KpnI**	CGCGCG**GGTACC**CAGGTTGAAGCGGGTGTTAG	*attC* site (reverse primer)	This study
**TMB4-Pc-F1-KpnI**	CGGCCG**GGTACC**TGCGTGCGTTATCCCATTTA	TMB4 Pc promoter (forward primer)	This study
**TMB4-Pc-R1-EcoRI**	CGCGCG**GAATTC**CATCTTTTCGACCTTTCCTC	TMB4 Pc promoter (reverse primer)	This study
**TMB4-GC-F1-KpnI**	CGCGCG**GGTACC**TTAGACAGATGCCTTGCGG	TMB4 gene cassette (forward primer)	This study
**TMB4-GC-F1-EcoI**	CGCGCG**GAATTC**TTAGACAGATGCCTTGCGG	TMB4 gene cassette (forward primer)	This study
**TMB1-F1-KpnI**	CGCCGG**GGTACC**CGATCTTTCTTTTTTCCGTT	TMB1 Pc promoter (forward primer)	This study
**TMB1-R1-EcoRI**	CCCGCC**GAATTC**ACTTCCCTTCGACCCTTCCT	TMB1 Pc promoter (reverse primer)	This study
**Pfla-A**	**C**GACTTTTTTCCTAAACCCGCCTTAAAAATAAGCCGAAAATTTATTGAAGTAACATAGGATCAATGTATAGGAGGTTCAT**G**	P_{fla} sense strand oligo	[3]
**Pfla-B**	**AATTC**ATGAACCTCCTATACATTGATCCTATGTTACTTCAATAAATTTTCGGCTTATTTTTAAGGCGGGTTTAGGAAAAAAGTC**GGTAC**	Pfla antisense strand oligo	[3]

Primer name	Sequence (5’-3’)[a]	Gene target	Reference
**Tdtrop/O-A**	**C**GGGATTCAGCTTGACATTTTCTTTATTTTTTTATATATTATAATCATAATTTTGATATATCAAAATAGGAGATTTGAA**G**	P_{TdTro} sense strand oligo	[4]
**Tdtrop /O-B**	**AATTC**TTCAAATCTCCTATTTTGATATATCAAAATTATGATTATAATATATAAAAAAATAAAGAAAATGTCAAGCTGAATCCC**GGTAC**	P_{TdTro} antisense strand oligo	[4]
**Primers for the construction of pCC1BAC-*lacZα-gusA* vectors.**			
***lacZ*-F1**	CGGCGC**GACGTC**AGAGAATATAAAAAGCCGGATTATTAATCCGGCTTTTTTATTATTTGTCGGGGCTGGCTTAACTAT	*lacZα* (Forward primer)	This study
***lacZ*-R1**	GCGGCG**ATGCAT**TGTGAGCGGATAACAATTTC	*lacZα* (Reverse primer)	This study
***gusA*-F1**	CGCGCG**ATGCATGCTAGC**ATCACGAATTCCTGCAGTAA	*gusA* (Forward primer)	This study
***gusA*-R1**	GGGCGG**CCTAGG**AAATAATAAAAAAGCCGGATTAATAATCCGGCTTTTTATATTCTCTCGCCAGGAGAGTTGTTGATT	*gusA* (Forward primer)	This study
**pCC1BAC-del*LacZ*-F1**	CCCCCC**CCTAGG**CCGTCGACCAATTCTCATGT	pCC1BAC *lacZα* deletion (Forward primer)	This study
**pCC1BAC-delLacZ-R1**	GCGGCG**GACGTC**TAGTTAAGCCAGCCCCGACA	pCC1BAC *lacZα* deletion (reverse primer)	This study
**Primers for the amplification of GCs and promoters into pCC1BAC-*lacZα-gusA* vectors.**			
**pUC-GC-F1-NsiI**	CGCGCG**ATGCAT**TTGTAATTCGACGGCCAGTG	GC on pUC19-GC-*gusA* construct (forward primer)	This study
**pUC-GC-R1-NheI**	GGGCGG**GCTAGC**ATTTTCTCCGCTACTCCAGG	GC on pUC19-GC-*gusA* construct (reverse primer)	This study

Primer name	Sequence (5'-3')[a]	Gene target	Reference
**pUC-GC-F1-NheI**	CGCGCG**GCTAGC**TTGTAATTCGACGGCCAGTG	GC on pUC19-GC-*gusA* construct (forward primer)	This study
**pUC-GC-R1-NsiI**	GGGCGG**ATGCAT**ATTTTCTCCGCTACTCCAGG	GC on pUC19-GC-*gusA* construct (reverse primer)	This study
**SUPA4-NsiI**	GCGGCC**ATGCAT**CCGCAAATGCAGGTTAAGCG	*attC* site (forward primer)	This study
**SUPA3-NheI**	CGCGCG**GCTAGC**CAGGTTGAAGCGGGTGTTAG	*attC* site (reverse primer)	This study
**MARS5-NsiI**	GCGGCC**ATGCAT**CGCAAATGCAGGTTAAGCG	*attC* site (forward primer)	This study
**MARS2-NheI**	CGCGCG**GCTAGC**GCAATGTCAGGTTGAAGC	*attC* site (reverse primer)	This study
**Primers for sequencing of inserts in pCC1BAC-*lacZα-gusA* vectors**			
***lacZ*-F2**	GTTTTCCCAGTCACGACGTT	Insert forward sequencing	This study
***gusA*-R2**	CGGCGAACTGATCGTTAAAA	Insert reverse sequencing	This study

[a] Restriction sites and bi-directional terminators were indicated as bold and italic styles, respectively.

Supplementary Table S4; Complementarity of the core sites R′ (1R) and R′′ (1L) abutting the forward and reverse attC primer sequence on the gene cassettes.

**Type (Number of GCs)**	**Sequence of R′ after the forward primer sequence of the *attC* on GCs**	**Pattern of R′ sequence of the GC[a]**	**Sequence of R″ before the reverse primer of the *attC* on GCs GC**	**Pattern of R″ sequence of the *attC* on GCs GC[a]**	**Gene cassettes**	**Complementarity between R′ and R″ core sites of the *attC* on GCs**
**A (15)**	GTTAGGT	GTTRRRY	ACCTAAC	RYYYAAC	SSU-Pro-20, SSU-Pro-32, SSU-Pro-65, MMU-PRO-24, MMU-PRO-48	7/7
	GTTAGAC	GTTRRRY	GTCTAAC	RYYYAAC	SSU-Pro-24, MMU-Pro-4 MMU-PRO-5, MMU-PRO-18, MMU-PRO-22, MMU-PRO-53	7/7
	GTTGAAC	GTTRRRY	CTTCAAC	YYYYAAC	SSU-Pro-9, SSU-Pro-34	6/7
	GTTAGAC	GTTRRRY	GCCTAAC	RYYYAAC	MMU-PRO-6	6/7
	GTTGAAC	GTTRRRY	GTCTAAC	RYYYAAC	MMU-PRO-31	5/7
**B (3)**	GTTAGAA	GTTRRRR	TTCTAAC	YYYYAAC	SSU-Pro-7	7/7
	GTTAGAG	GTTRRRR	CTCTAAC	YYYYAAC	SSU-Pro-46	7/7
	GTTAGGA	GTTRRRR	TCCTAAC	YYYYAAC	MMU-PRO-65	7/7

**Type (Number of GCs)**	**Sequence of R′ after the forward primer sequence of the *attC* on GCs**	**Pattern of R′ sequence of the GC[a]**	**Sequence of R″ before the reverse primer of the *attC* on GCs GC**	**Pattern of R″ sequence of the *attC* on GCs GC[a]**	**Gene cassettes**	**Complementarity between R′ and R″ core sites of the *attC* on GCs**
**C (3)**	GTTATGT	GTTRYRY	ACCTAAC	RYYYAAC	SSU-Pro-16	6/7
	GTTATGT	GTTRYRY	ACCTAAC	RYYYAAC	SSU-Pro-39	6/7
	GTTATAC	GTTRYRY	GCCTAAC	RYYYAAC	MMU-PRO-63	5/7
**D (2)**	GTTAGCT	GTTRRYY	AGCTAAC	RRYYAAC	SSU-Pro-13	7/7
	GTTAGCT	GTTRRYY	ATCTAAC	RYYYAAC	SSU-Pro-27	6/7

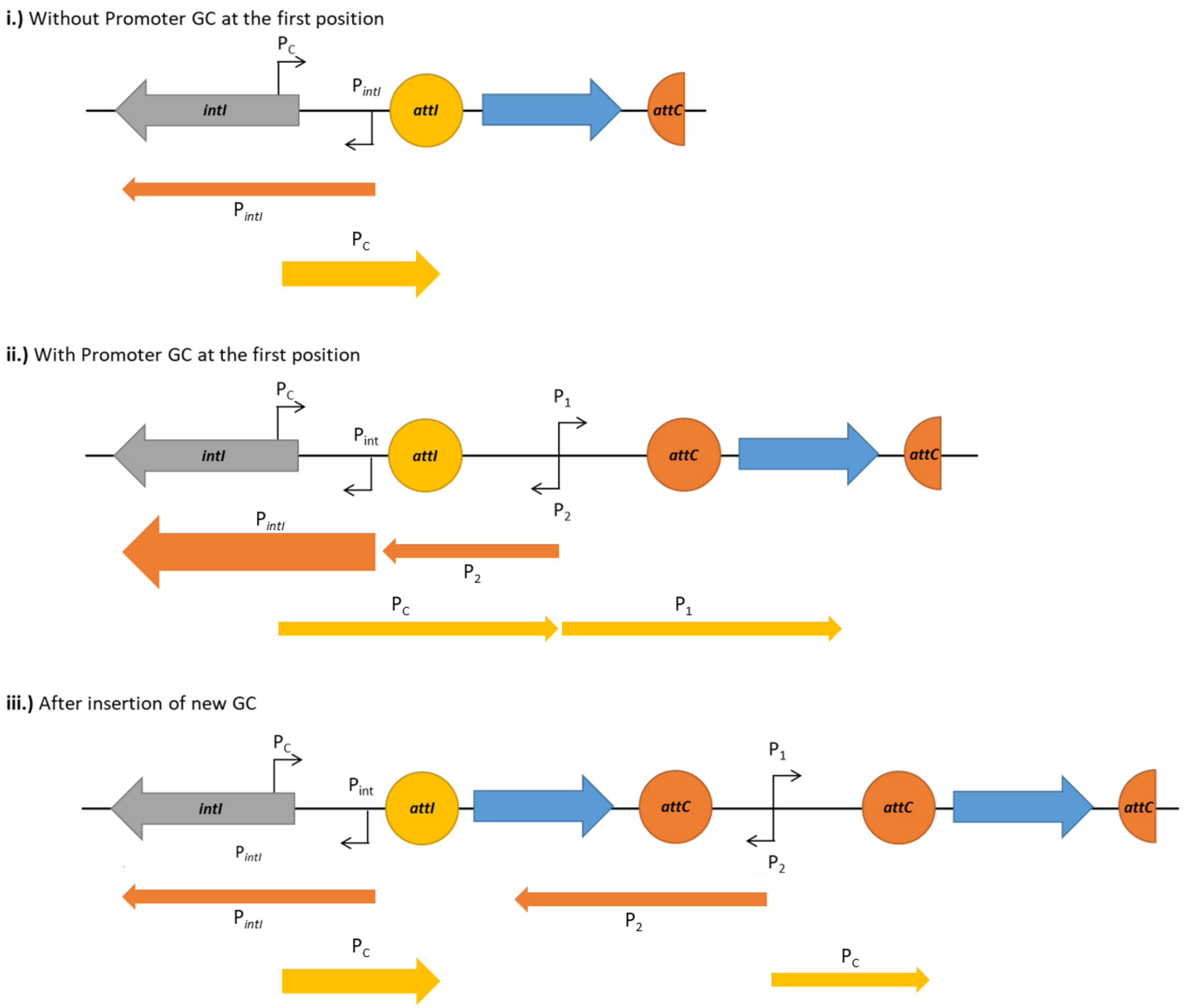

Supplementary Figure 1: The proposed hypothesis regarding integrase expression when the promoter GC is inserted in the first position of in integron. (i) Without the promoter GC, it was previously shown that there was a transcriptional interference (TI) between P_{intI} and P_C which controls the expression of integrase[5,6]. (ii) The insertion of promoter GC in the first position would increase the expression of integrase, as the antisense GC promoter (P_2) could increase transcription on the antisense strand and relieve the TI between P_{intI} and P_C by reducing activity at Pc. (iii) Increased expression of integrase would catalyse the insertion of a new GC at the *attI,* shifting the promoter GC into the second position. The level of integrase will be decreased as there will be lower de-repression of TI from the antisense promoter GC. The grey and blue open arrowed boxes represent integrase gene (*intI*) and the open reading frames (ORFs), respectively, pointing in the direction of transcription. The recombination sites, *attI* and *attC*, were represented by yellow and orange circles, respectively. The yellow and orange

arrows indicate the promoter activity in sense and antisense strands, respectively. The thickness of the arrows represents the hypothesised relative promoter activity in each stage.

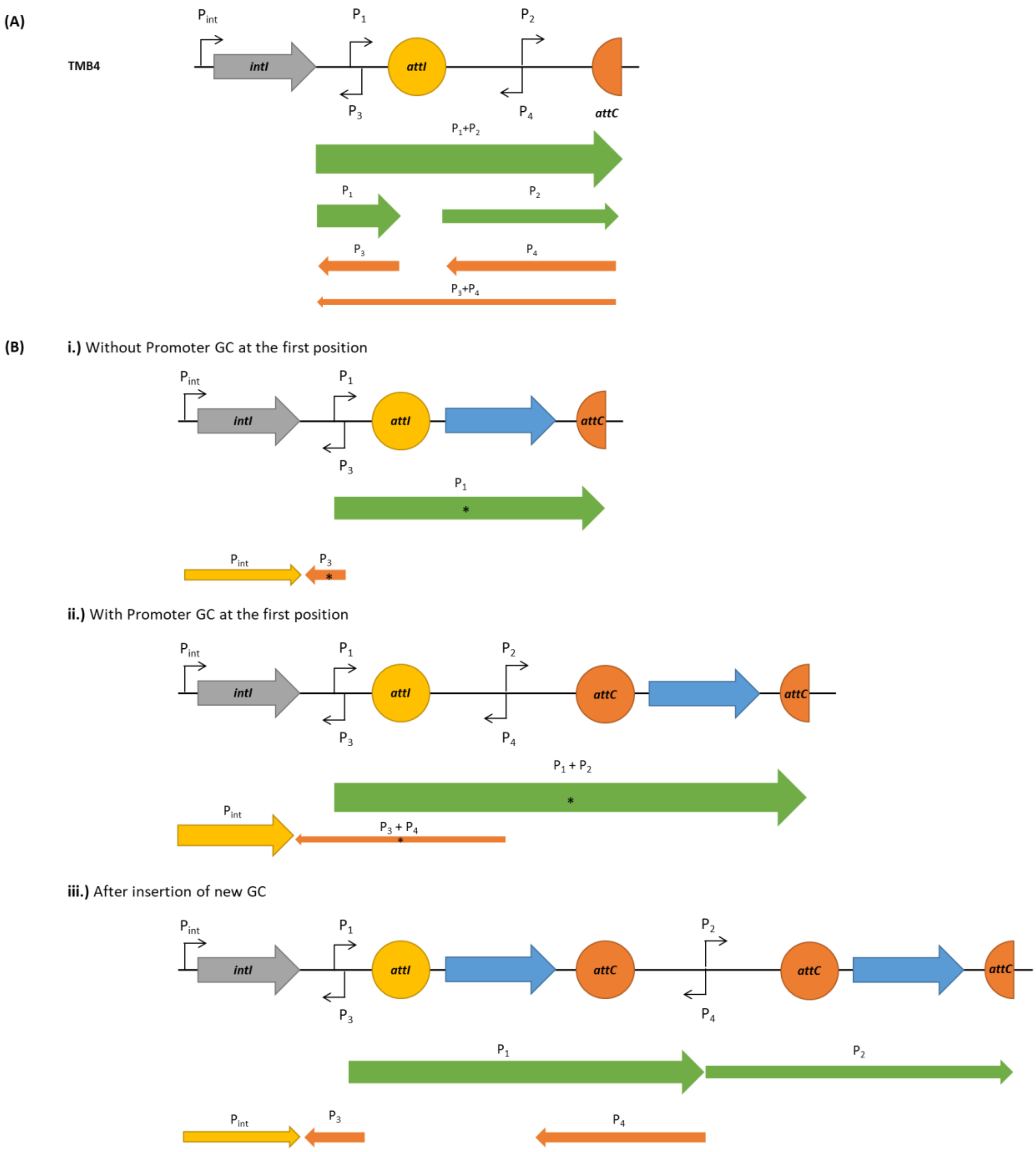

Supplementary Figure 2: The transcriptional activity (TI) of the TMB4 GC constructs. (A) Schematic representation of the structure of TMB4 in the integron. The thickness of the arrows represents the relative promoter activity from β-glucuronidase assays in TMB4 P_C (P_1 and P_3), GC (P_2 and P_4) and P_C+GC (P_1+P_2 and P_3+P_4), based on the results in Figure 4. (B) The proposed hypothesis on integrase expression when the promoter GC is inserted in the first position of in the reverse integrons. (i) Without the promoter GC, there are promoter activities in both sense and antisense direction from P_C (P_1 and P_3) as shown by the results in TMB4 P_C in figure 4. (ii) When the promoter GC inserted in the first position,

the expression level on the sense strand increased (P_1 + P_2), which in turn decreased the expression level on the antisense strand (P_1+P_3), possibly through TI, as shown by the activity of TMB4 P_C+GC in Figure 4. The level of P_{intI} is, therefore, hypothesised to be increased, due to the de-repression of TI between P_{intI} and P_3 by the insertion of the promoter GC. (iii) Increasing integrase expression will catalyse an insertion of a new GC at *attI*, which will push the promoter GC the second position. This would reduce the expression level of integrase because the promoter activity of P_3 would be restored and increased due to an absence of TI from P_2, resulting in higher TI between P_{intI} and P_3. The grey and blue open arrowed boxes represent integrase gene (*intI*) and the open reading frames (ORFs), respectively, pointing in the direction of transcription. The recombination sites, *attI* and *attC*, were represented by yellow and orange circles, respectively. The green and orange arrows indicate the promoter activity from the P_C promoter and promoter GC in sense and antisense strands, respectively, while the yellow arrows represent the promoter activity from P_{intI}. The asterisks indicate the experimentally verified relative expression level, shown in Figure 4 (TMB4 P_C and TMB4 P_C+GC).

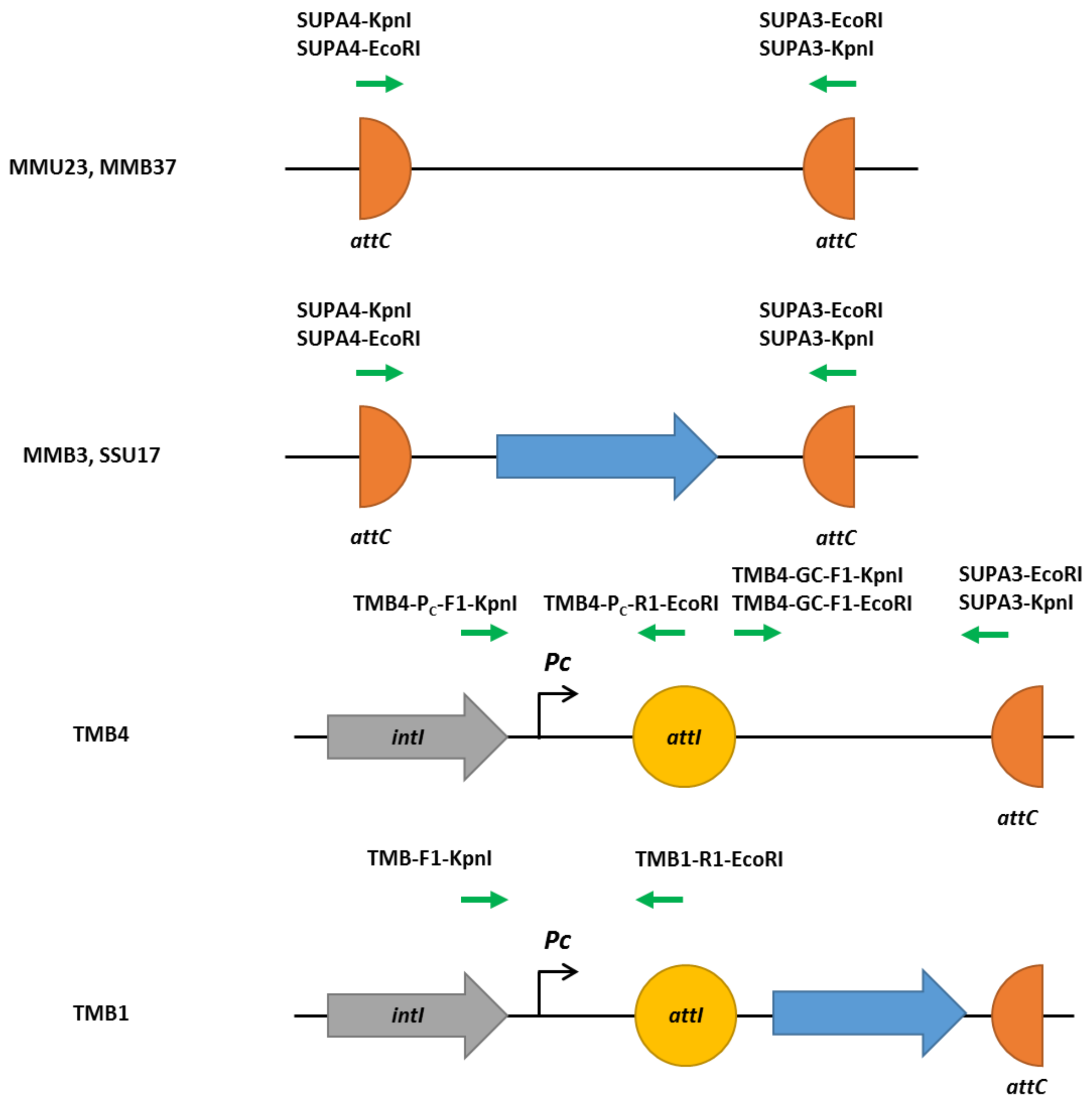

Supplementary Figure 3: The amplification of the selected GCs from GC-containing pGEM-T easy vectors. The green arrows indicate the primer binding sites. The grey and blue open arrowed boxes represent integrase gene (*intI*) and the open reading frames (ORFs), respectively, pointing in the direction of transcription. The promoters, P_{intI} and P_C, were represented by black arrows. The recombination sites, *attI* and *attC*, were represented by yellow and orange circles, respectively.

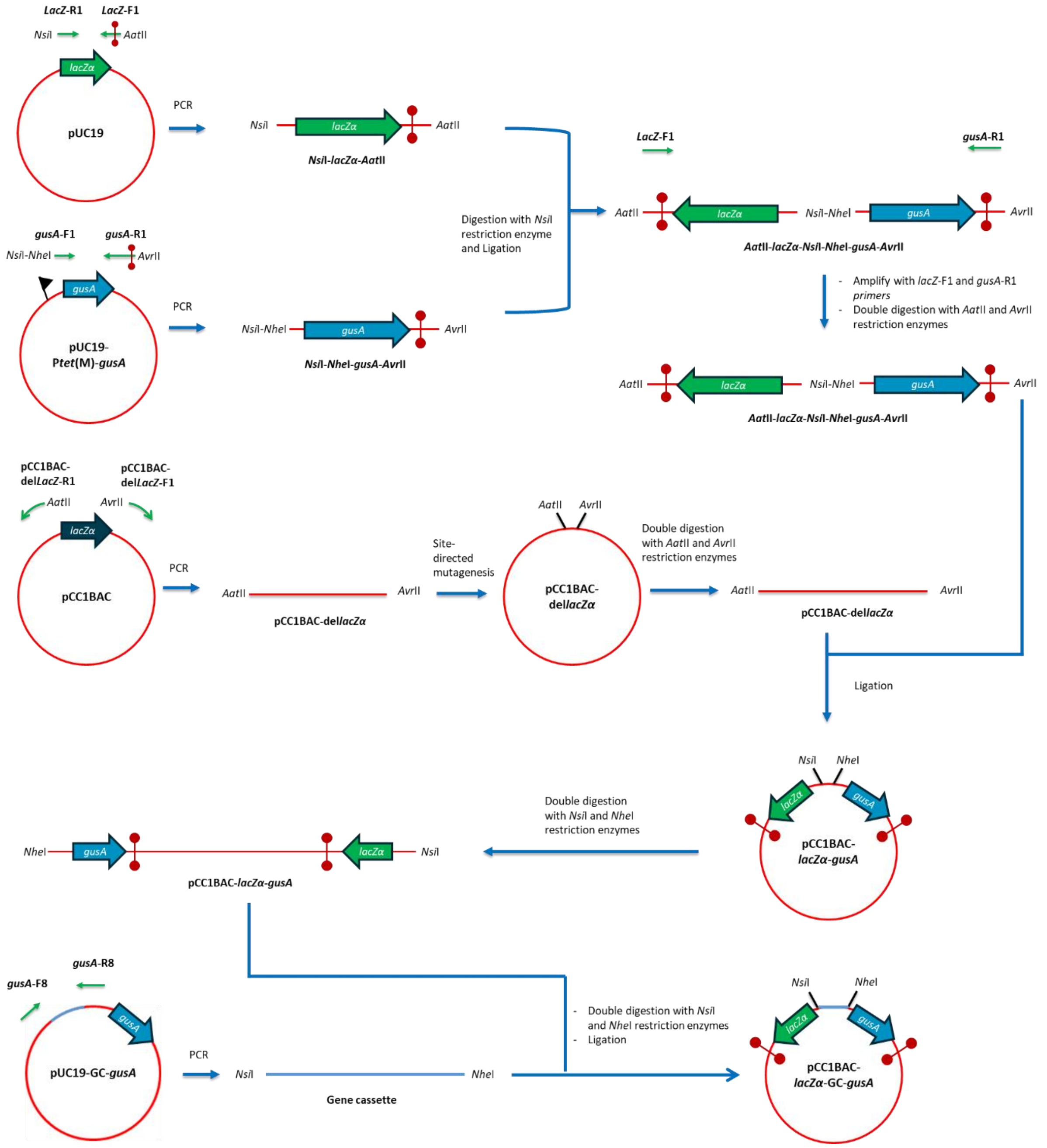

Supplementary Figure 4: Construction of pCC1BAC-*lacZα*-GC-*gusA* constructs. The *lacZα* and *gusA* reporter genes were amplified from pUC19 and pUC19-P*tet*(M), respectively. Both reporters were ligated together and inserted in between *Aat*II and *Avr*II sites of the pCC1BAC-del*lacZα* plasmid, forming pCC1BAC-*lacZα-gusA*. The inserts were amplified and cloned into the *Nsi*I and *Nhe*I sites on pCC1BAC-*lacZα-gusA* plasmid. The green arrows and black lines represent primer binding sites and restriction sites, respectively. The symbol ( ) and ( ) represent promoter and bi-directional

terminators, respectively. The reporter genes were represented as open arrow boxes, pointing in the direction of transcription.

References

1 Solovyev, V. & Salamov, A. in *In Metagenomics and its Applications in Agriculture, Biomedicine and Environmental Studies* (ed R.W. Li) 61-78 (Nova Science Publishers, 2011).

2 Tansirichaiya, S., Rahman, M. A., Antepowicz, A., Mullany, P. & Roberts, A. P. Detection of Novel Integrons in the Metagenome of Human Saliva. *PloS one* **11**, e0157605, doi:10.1371/journal.pone.0157605 (2016).

3 Limberger, R. J., Slivienski, L. L., Izard, J. & Samsonoff, W. A. Insertional inactivation of *Treponema denticola tap1* results in a nonmotile mutant with elongated flagellar hooks. *Journal of bacteriology* **181**, 3743-3750 (1999).

4 Brett, P. J., Burtnick, M. N., Fenno, J. C. & Gherardini, F. C. *Treponema denticola* TroR is a manganese- and iron-dependent transcriptional repressor. *Molecular microbiology* **70**, 396-409, doi:10.1111/j.1365-2958.2008.06418.x (2008).

5 Jove, T., Da Re, S., Denis, F., Mazel, D. & Ploy, M. C. Inverse correlation between promoter strength and excision activity in class 1 integrons. *PLoS genetics* **6**, e1000793, doi:10.1371/journal.pgen.1000793 (2010).

6 Guerin, E., Jove, T., Tabesse, A., Mazel, D. & Ploy, M. C. High-level gene cassette transcription prevents integrase expression in class 1 integrons. *Journal of bacteriology* **193**, 5675-5682, doi:10.1128/jb.05246-11 (2011).
